# In Vivo Assessment of Individual and Total Proteinuria in Zebrafish Larvae Using the Solvatochromic Compound ZMB741

**DOI:** 10.1021/cbmi.4c00029

**Published:** 2024-05-31

**Authors:** Tsuyoshi Nomoto, Aoi Mori, Kayoko Yamada, Fumihiro Terami, Akiyoshi Shimizu, Toshio Tanaka

**Affiliations:** † Department of Systems Pharmacology, Mie University Graduate School of Medicine, Tsu, Mie 514-8507, Japan; ‡ Mie University Medical Zebrafish Research Center, Tsu 5148572, Japan

**Keywords:** zebrafish, proteinuria, pronephric tubule, solvatochromic dye, pathophysiological screening

## Abstract

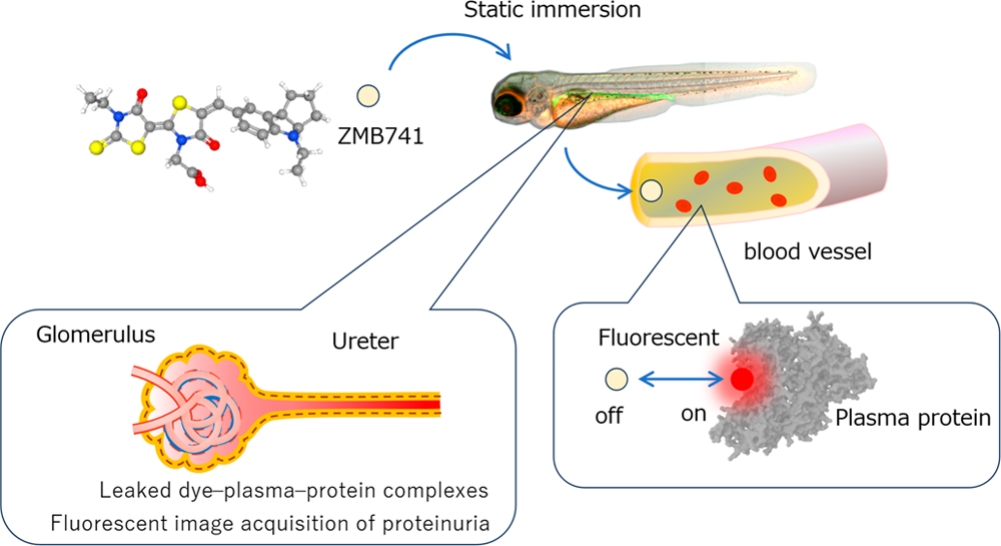

The robustness of blood filtration in the kidney is supported by two major functions: the molecular sieve of the glomerulus and reabsorption of the proximal tubules. Detecting glomerular dysfunction is challenging because of the compensatory nature of proximal tubule reabsorption. To facilitate pathophysiological studies of the vertebrate kidney, zebrafish pronephroi are used, owing to their simple glomerular and proximal tubular configuration. In this study, a solvatochromic dye with an affinity for plasma proteins was used to detect urinary proteins leaking into the ureter of zebrafish. Aristolochic acid exposure to fertilized eggs of transgenic zebrafish expressing green fluorescent protein from the proximal tubules to the excretory pore induced concentration-dependent renal dysfunction. The solvatochromic dye ZMB741 was applied via static immersion to analyze leaked dye–plasma–protein complexes in the ureter; their axial distribution was imaged by using confocal microscopy. The effect of resveratrol, an attenuator of aristolochic acid nephropathy, was further analyzed. This method enables individual-level analysis of podocytopathy, a mild glomerular disease that does not necessarily lead to the excretion of proteinuria. Moreover, it will be useful for pathophysiological studies of renal function and the identification of potential therapeutic drugs.

## Introduction

Proteinuria is a marker and risk factor for kidney disease progression.^1^ The development of proteinuria involves two major mechanisms: increased permeability of the glomerular capillary wall^2^ and impaired protein reabsorption in the proximal tubules.^3^ Under physiological conditions, the glomerular capillary wall restricts the filtration of plasma proteins based on their molecular size, charge, and steric structure. Although low-molecular-weight plasma proteins and some medium-molecular-weight plasma proteins pass through the glomerular wall, they are entirely reabsorbed by multiligand receptors in proximal tubular cells, preventing their appearance in urine. Reabsorption in the proximal tubule is dependent on the type, size, and charge of the protein. However, as structural disruption of the glomerular capillary wall progresses, medium- and high-molecular-weight proteins are abnormally filtered out of the glomerulus. This causes excessive burden and toxicity to tubular epithelial cells, leading to a reduction in the reabsorption of low-molecular-weight proteins in the proximal tubules and their excretion in urine. The distribution of protein molecular weight in urine exhibits varied molecular profiles, which depend on the permeability of the glomerular capillary wall and the degree of proximal tubule dysfunction. Molecular weight distributions of urinary proteins aid in diagnosing different types of kidney disease,^3^ including diabetic nephropathy,^6^ chronic kidney disease,^7^ and glomerulonephritis.^8^ Molecular weight distributions can also be used to monitor the progression of kidney disease and to assess the response to treatment.^4,5^

The zebrafish pronephros, with its simple and functional segmentation, is a favorable model to study mammalian nephrons, as they share several similarities.^9,10,11,12^ Based on the mapping of expressed genes, it has been observed that the pronephric duct, previously thought to be a uniform duct, is actually composed of multiple segments, including the proximal convoluted tubule, proximal straight tubule, distal early tubule, corpuscle of Stannius, and distal late tubule.^11^

One method for assessing glomerular injury in zebrafish involves injecting them with fluorescent-labeled high-molecular-weight dextran as an exogenous tracer.^13,14^ Alternatively, transgenic zebrafish lines have been created with fusion proteins of vitamin D binding protein and enhanced green fluorescent protein (GFP)^15,16^ or vitamin D binding protein and nanoluciferase, serving as endogenous tracers for this purpose.^17^ Traditional tracers are single-molecular-weight substances that differ from the original plasma proteins, limiting their ability to accurately infer renal dysfunction from tracer leakage.

The purpose of this study was to demonstrate that solvatochromic dyes, which have an affinity for plasma proteins, can be used to visualize the distribution of native plasma proteins that leak into the ureter after kidney injury in larval zebrafish. One such solvatochromic dye compound, ZMB741, rapidly enters the bloodstream of zebrafish via static immersion and increases fluorescence intensity based on its binding affinity for plasma proteins.^18^ ZMB741 was initially identified as a dye with an affinity for albumin;^18^ however, as ZMB741 can strongly fluoresce the blood of zebrafish despite the absence of albumin,^19^ the affinity and fluorescence enhancement of ZMB741 was confirmed using a commercially available protein homologous to the major plasma protein of zebrafish. Aristolochic acid damages both glomeruli and tubules in zebrafish larvae.^20^ The appropriateness of using aristolochic nephropathy as a model system for proteinuria with different levels of molecular weight distributions was validated in this study. Resveratrol, an attenuator of aristolochic acid nephropathy,^21^ was used to explore the possibility of discovering candidate compounds for therapeutic agents using the methods of this study. Our innovative approach of visualizing the clearance of intrinsic leaky plasma proteins in the ureter from the proximal tubule to the excretory pore at the individual level may be a useful tool for the study of the pathophysiology of renal dysfunction and the development of therapeutic agents.

## Materials and Methods

### Ethical Approval

This study adhered to the ethical guidelines established by the Institutional Animal Care and Use Committee of Mie University, Japan.

### Zebrafish Strains

A zebrafish mutant line, MieKomachi 067 (MK067), was generated by crossing Tg (enpep:GFP)^22^ and MieKomachi 001 (MK001).^23^ MK067 exhibited transparency and expressed GFP from the pronephric glomeruli to the excretory pore. Embryos were obtained via natural mating and cultured at 28.5 ± 0.5 °C with a light/dark cycle of 14 h light and 10 h dark in 0.3× Danieau’s solution [19.3 mM NaCl, 0.23 mM KCl, 0.13 mM MgSO_4_, 0.2 mM Ca(NO_3_)_2_, 1.7 mM HEPES, pH = 7.2] until 5 days post-fertilization.

### Compounds

Aristolochic acid I (AA) was acquired from Sigma-Aldrich (St. Louis, Missouri, U.S.A.). Resveratrol was procured from Tokyo Chemical Industry (Tokyo, Japan). ZMB741 was obtained from Canon Inc. (Tokyo, Japan). These compounds were dissolved in dimethyl sulfoxide (DMSO; Nacalai Tesque, Kyoto, Japan) to prepare stock solutions. Human apolipoproteins A-I and human plasma hemopexin were purchased from Sigma-Aldrich.

### Induction of Aristolochic Acid Nephropathy

Embryos of the MK067 strain were incubated in 0.3× Danieau’s solution containing AA for 5 h (24–29 h post-fertilization, 5 or 20 μM AA, 0.25 v/v% DMSO). Embryos from the control group were incubated in 0.3× Danieau’s solution containing the vehicle (0.25 v/v% DMSO). At 3 days post-fertilization, larvae with 200 μL of culture water were individually transferred to each well of a 96 multiwell plate. Immediately thereafter, 100 μL of culture water was collected as an initial urine sample. The remaining 100 μL was collected for storage after 24 h of incubation at 28 °C. Each sample was lyophilized and dissolved in 10 μL of 30 mM Tris-HCl buffer (pH = 8.5). Then, 5 μL of the concentrated sample was processed under reducing conditions using the High Sensitivity Protein 250 Kit (Agilent Technologies, Santa Clara, California, U.S.A.) with the additional Pico protocol specified by the manufacturer (Agilent Technical Note, publication no. 5990-3703EN). Each sample was analyzed using an Agilent 2100 Bioanalyzer instrument (Agilent Technologies). To compare the graph area of the electropherogram with the protein concentration, the value of the “time-corrected area”/“area” of the simultaneously analyzed ladder peak was approximated using a power function regarding the “aligned migration time”. The approximate value was multiplied to calculate the “time-corrected value”. Data processing was performed using Microsoft Excel based on a CSV file.

### Measurement of Fluorescence Spectrum and Intensity

Fluorescence excitation, emission spectra, and intensities of ZMB741 solutions were acquired by using a Spark multimode microplate reader with the SparkControl software (Tecan, Männedorf, Switzerland). Measurements of various concentrations of ZMB741 solutions, with or without the target protein, were performed in Greiner 384 flat transparent plates (Greiner Bio-One, Kremsmünster, Austria).

### Affinity Measurement of ZMB741 for the Target Protein

Quantification of the affinity parameters that define the formation of chemical species responsible for the fluorescence enhancement in the system, where the target protein coexists with ZMB741, was estimated using nonlinear multiple regression with binding eq 1:
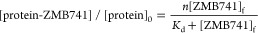
1where [protein-ZMB741] represents the concentrations of the chemical species in the fluorescence-enhanced state, [protein]_0_ is the initial amount of the target protein, [ZMB741]_f_ is the concentration of ZMB741 in the free state, *n* is dimensionless and corresponds to the maximum binding number, and *K*_d_ is the dissociation constant. Under a constant amount of target protein and varying amounts of ZMB741, concentrations of the chemical species ([protein-ZMB741]) were determined by using fluorometry. The conversion coefficient (α) of the fluorescence intensity versus the concentration of the chemical species ([protein-ZMB741]) was tentatively obtained as follows: the fluorescence intensities of systems containing multiple concentrations of target proteins coexisting with a constant low concentration of ZMB741 were measured. The fluorescence intensities for 1/[protein]_0_ were approximated via a linear relationship, and the *y*-intercept was estimated by extrapolation to represent the fluorescence enhancement for an excess amount of target protein. Nonlinear multiple regression was performed using Microsoft Excel (Microsoft Co., U.S.A.).

### Fluorescent Staining of Proteins in Urinary Duct and Acquisition of Fluorescent Images

Zebrafish larvae were immersed statically in 0.3× Danieau’s solution containing ZMB741 (1 μM, 1 v/v% DMSO) at 28.5 °C for 3 h. Thereafter, larvae were anesthetized with 500 ppm of 2-phenoxyethanol and individually transferred to a 96-well format ZF plate (Hashimoto Electronic Industry, Mie, Japan) with a 0.17 mm glass bottom, using 75 μL of staining solution per well. To ensure proper distribution of the larvae, brief centrifugation was performed (200*g*, 10 s), followed by manual placement of the larvae in the right lateral position. The volume of the staining solution in each well was subsequently reduced to 25 μL. Confocal *z*-stack images of the larvae were acquired by using a 10× objective and a CQ1 confocal microscope (YOKOGAWA CQ1, Japan) with a *z*-stack interval of 3.947 μm. The CQ1 image acquisition process commenced 6 h after staining initiation, and images were acquired over a period of 4 h and 40 min, maintaining the same staining conditions.

### Fluorescent Image Analysis of Leaked Plasma Proteins in Zebrafish Pronephros

**Figure 1:**
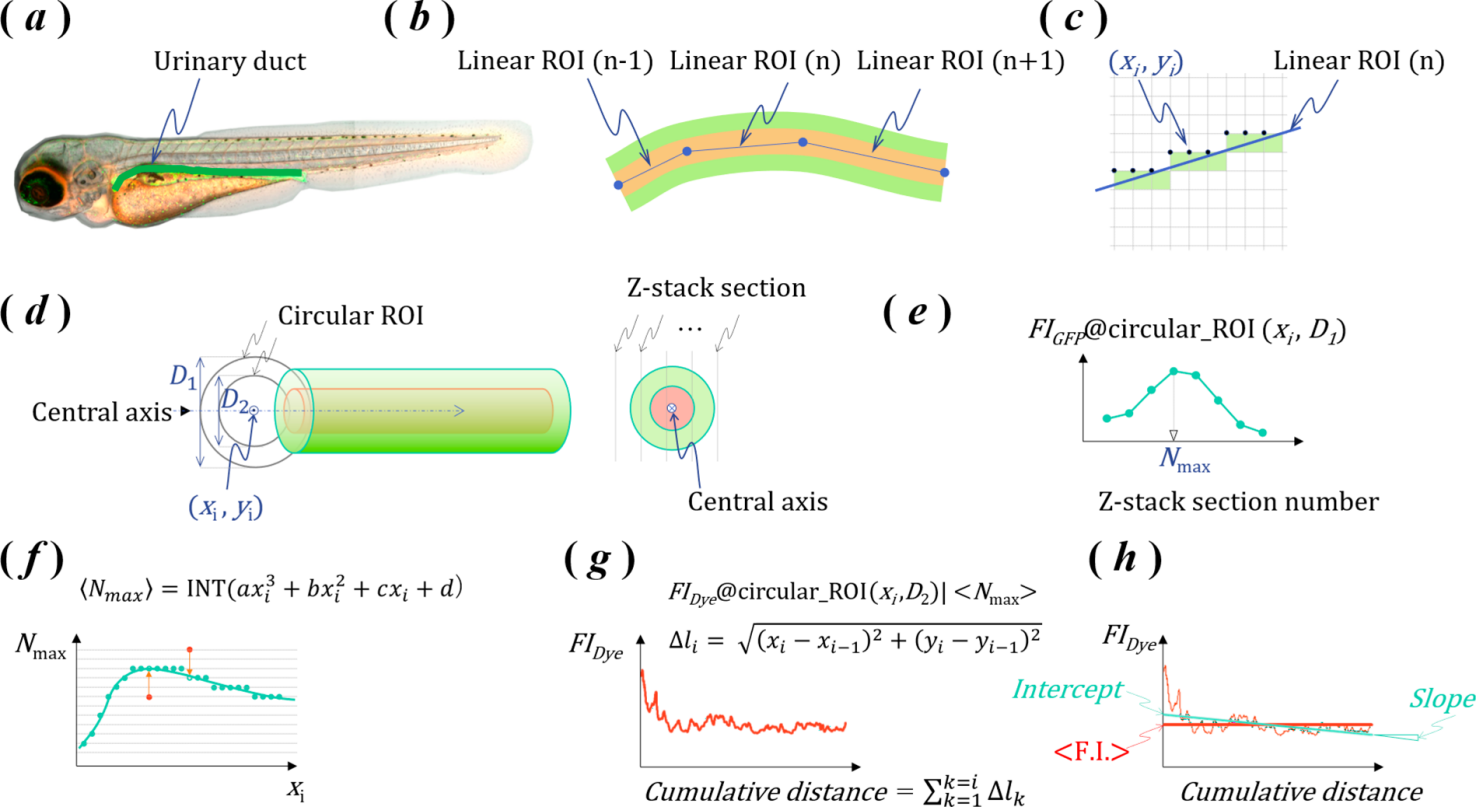
Fluorescent image analysis procedure for leaked plasma proteins in zebrafish pronephros. (a) Urinary duct from the GFP MIP image. (b) Urinary duct axis as a collection of straight-line ROIs. (c) Pixels [integer coordinate values (*x*_*i*_, *y*_*i*_)] on the straight-line ROIs. (d) A circular ROI (*x*_*i*_, *D*_1_) with a diameter *D*_1_ and a circular ROI (*x*_*i*_, *D*_2_) with a diameter *D*_2_. (e) Optical slice (section number) *N*_max_ where the fluorescence intensity FI_GFP_ at circular ROI (*x*_*i*_, *D*_1_) is the maximum. (f) Plot showing *N*_max_ for all *x*_*i*_ corrected for jumps in *N*_max_ values by approximating them with a cubic function. (g) Dye fluorescence intensity of the circular ROI (*x*_*i*_, *D*_2_) at the optical slice ⟨*N*_max_⟩ used as the intraureteric dye fluorescence intensity at coordinates (*x*_*i*_, *y*_*i*_). The horizontal axis represents the cumulative distance ∑_*k* = 1_^*k* = *i*^Δ*l*_*k*_, which is the accumulated value of the distances  between the centers of adjacent circular ROIs. (h) Average transition of FI_dye_ against the cumulative distance, fluorescence intensity, and the intercept and slope of the linear approximation.

The procedure for the fluorescence image analysis of leaked plasma proteins in the zebrafish pronephros is summarized in Figure 1. Maximum intensity projection (MIP) images were generated from confocal *z*-stack images, and based on the fluorescence image of GFP, the ureteral portion from the proximal tubule to the excretory foramen was designated using multiple linear regions of interest (ROIs) along its axis. The coordinates (*x*_*i*_, *y*_*i*_) of all pixels through which the linear ROI passed were calculated. For all (*x*_*i*_, *y*_*i*_), a circle with a diameter (*D*_1_) centered at (*x*_*i*_, *y*_*i*_), encompassing the outer circumference of the ureter, and a circle with a diameter (*D*_2_), encompassing the inner diameter of the ureter, were defined as new circular ROIs (*x*_*i*_, *D*_1_) and (*x*_*i*_, *D*_2_), respectively. For all *z*-stack images, the GFP fluorescence intensity (FI_GFP_) at the circular ROI (*x*_*i*_, *D*_1_) was compared to determine the *z*-stack number [*N*_max_(*x*_*i*_)] with the maximum fluorescence intensity. The transition of *N*_max_(*x*_*i*_) with respect to *x*_*i*_ was approximated by a cubic function of *x*_*i*_ as well as the integer value ⟨*N*_max_(*x*_*i*_)⟩ closest to that calculated by substituting *x*_*i*_ into this approximation. Dye fluorescence intensity of the circular ROI (*x*_*i*_, *D*_2_) at *z*-stack number ⟨*N*_max_(*x*_*i*_)⟩ was obtained. The distance Δ*l*_*i*_ from the neighboring pixels, accumulated from the head side, was defined as the cumulative distance, and the dye fluorescence intensity (FI_dye_) of the ROI (*x*_*i*_, *D*_2_) was plotted against the cumulative distance. The average value of fluorescence intensity over the entire ureter was calculated. The slope and intercept were obtained via the linear approximation of the transition of FI_dye_ versus the total distance.

### Administration of Resveratrol

Resveratrol, an antagonist of AA nephropathy, was administered to zebrafish embryos prior to AA exposure following previously reported methods.^21^ Zebrafish embryos were statically immersed in 90 μM resveratrol from 12 to 24 h post-fertilization.

### Statistical Analyses

All statistical analyses were performed using R version 4.1.2 (R Foundation for Statistical Computing, Vienna, Austria). *P* < 0.05 was considered significant. Intergroup comparisons of AA exposure concentrations were analyzed using the Kruskal–Wallis/Bonferroni-adjusted rank sum test.

## Results and Discussion

**Figure 2:**
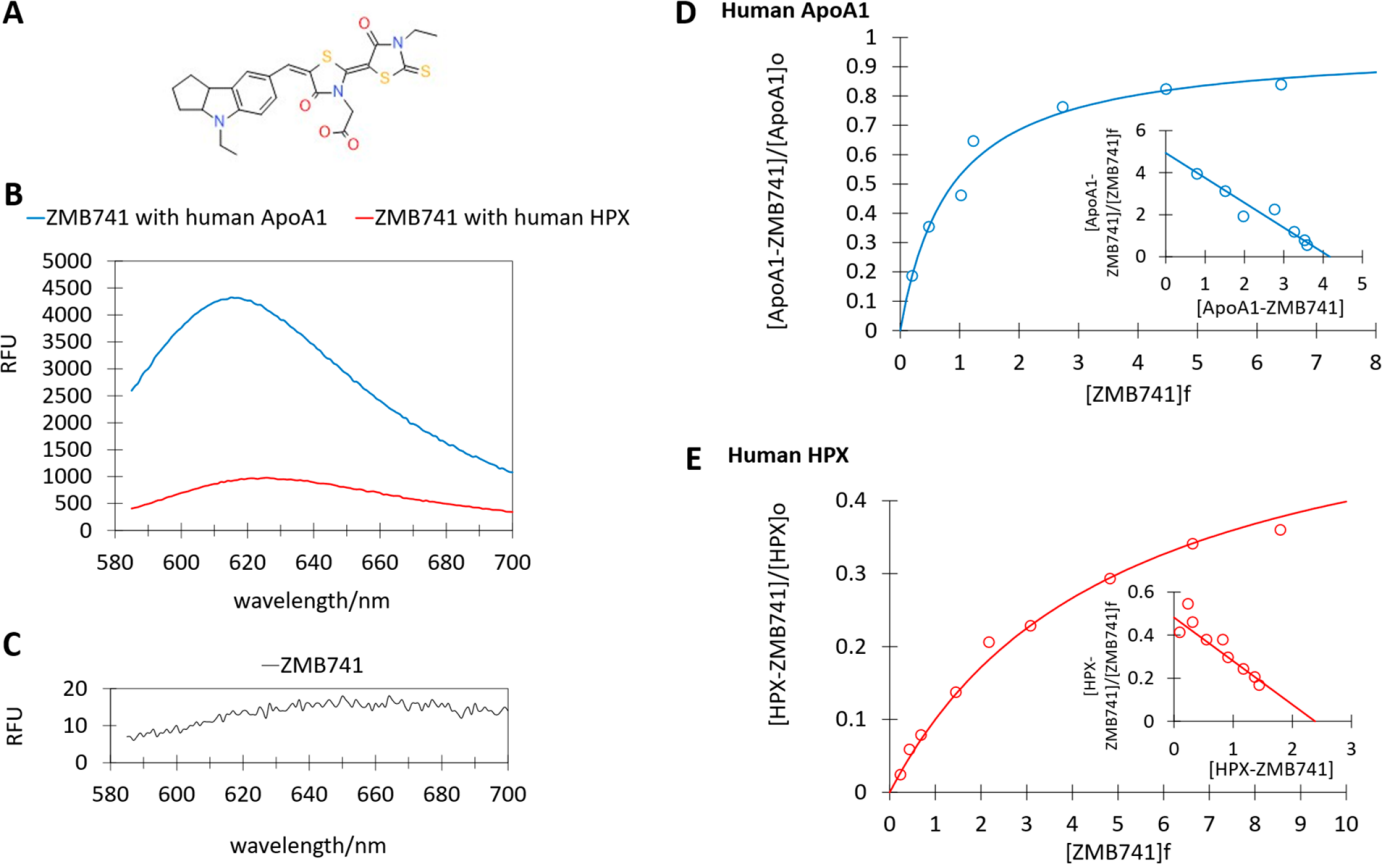
(A) Chemical structure of ZMB741. (B) Fluorescence spectra of 10 μM ZMB741 in PBS with 5.35 μM human ApoA1 or 10 μM human HPX. (C) Fluorescence spectra of 10 μM ZMB741 in PBS. (D, E) Binding properties of human ApoA1 and HPX. A Scatchard plot is shown for each binding curve.

ZMB741 (Figure 2A) was identified as a compound with an affinity for albumin.^18^ Despite the absence of albumin in the plasma of zebrafish,^24^ a strong fluorescence enhancement of ZMB741 by zebrafish blood components was observed.^18^ To estimate the target protein contributing to the fluorescence enhancement of ZMB741, we evaluated the affinity of ZMB741 for commercially available proteins homologous to major plasma proteins of zebrafish.^19^ According to BLASTP,^25,26^ the homology between human apolipoprotein A-I (NCBI-ProteinID: KAI2562951.1, 267aa) and zebrafish apolipoprotein A-Ib precursor (NCBI-ProteinID: NP_001093614.2, 257aa) was as follows: score, 63.5 bits (153); expectation, 8.00 × 10^–11^; identities, 50/176 (28%); positives, 84/176 (47%); and gaps, 16/176 (9%). The homology between human (NCBI-ProteinID: NP_000604.1, 462aa) and zebrafish (NCBI-ProteinID: XP_005173505.1, 447aa) hemopexin was as follows: score, 297 bits (761); expectation, 2.00 × 10^–95^; identities, 168/465 (36%); positives, 245/465 (52%); and gaps, 45/465 (9%). As shown in Figure 2 and Table 1, ZMB741 exhibited an affinity for the human plasma proteins apolipoprotein A-I and hemopexin, and upon binding, the fluorescence intensity increased. The maximum fluorescence enhancement ratio of ZMB741, when bound to the protein represented by α, was found to be in the range of 200- to 700-fold. A highly specific structural motif may not be required for the affinity protein to induce the strong fluorescence enhancement of ZMB741. This low specificity of ZMB741 could be used to detect nonspecific plasma-derived proteins in urine owing to podocyte injury.

**Table 1:** Affinity Parameters of ZMB741 with Commercially Available Proteins Homologous to the Major Plasma Proteins of Zebrafish

		kinetic parameters and fluorescence enhancement of ZMB741		
homologous protein	NCBI-ProteinID	*K*_d_ (μM)	*n* (-)	α (-)
apolipoprotein A-I human	KAI2562951.1	0.84	0.97	777
hemopexin from human plasma	NP_000604.1	3.13	0.54	223

### Efficacy of AA-Exposed Zebrafish as a Proteinuria Pathophysiology Model

**Figure 3:**
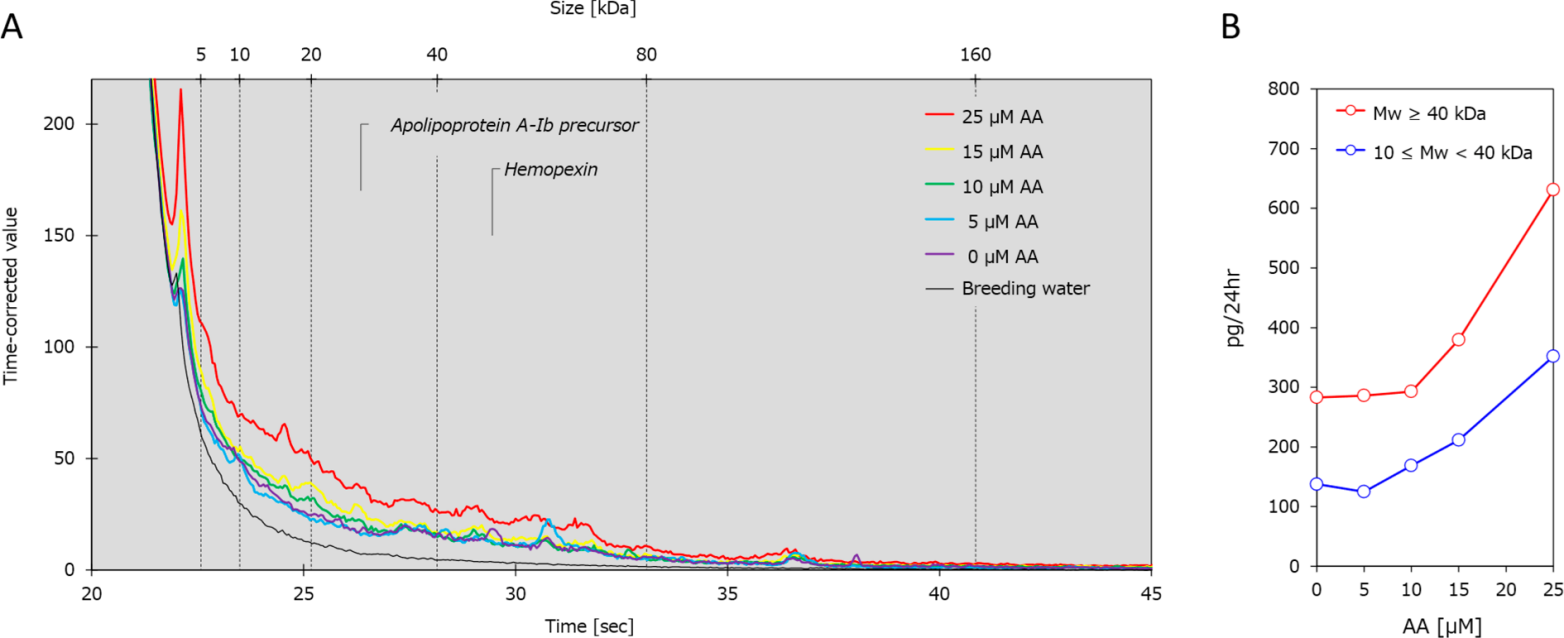
(A) Electropherograms of urine samples from zebrafish larvae exposed to AA at various concentrations. The vertical axis is corrected for retention time such that the interval integral with respect to time is proportional to the amount of protein. (B) Molecular weight distribution of urinary protein in larval zebrafish in response to AA exposure concentrations.

AA-exposed zebrafish demonstrated protein leakage that correlated with AA exposure concentrations (Figure 3A,B). The eluted proteins showed no specificity and consisted of a mixture of different molecular weights. Notably, no elution peaks corresponded to the molecular weights of the major plasma proteins of the zebrafish. This finding suggests the possibility of nonspecific leakage resulting from compromised integrity of the glomerular physical barrier. These results validate the use of AA-exposed zebrafish as a positive control in the proteinuria pathophysiology model used in this study.

The integrated area between the electropherogram of each sample and that of breeding water over a given molecular weight range was compared to the area of the ladder marker to estimate the amount of protein eluted in 24 h. When individuals were exposed to 5 μM AA concentrations, no significant increase in leakage was observed compared with the control group. In individuals exposed to 10 μM AA concentrations, a marginal increase in the leakage of low-molecular-weight proteins (10 kDa < MW < 40 kDa) was observed. Individuals exposed to high AA concentrations exhibited increased protein leakage, regardless of molecular weight. This observation suggests that the severity of AA nephropathy varies with exposure concentration. At low exposure concentrations, urinary protein appears with leakage of low-molecular-weight plasma proteins; as the exposure concentration increases, the severity of the disease increases, leading to leakage of high-molecular-weight plasma proteins.

Although some studies have used pooled samples from numerous individuals (e.g., ref (27)), the validity of using AA-exposed zebrafish for the development of proteinuria was investigated, as this model has not been used to demonstrate symptoms of proteinuria at the individual level. The increase in the amount of leaked protein correlated with AA exposure concentration, lack of leaked protein specificity, and consistency regarding the total amount of leakage, which led us to conclude that the model can be used to validate the feasibility of diagnosing proteinuria using dye.

### Comparing Aristolochic Acid Exposure Conditions and Ureter Distribution

**Figure 4:**
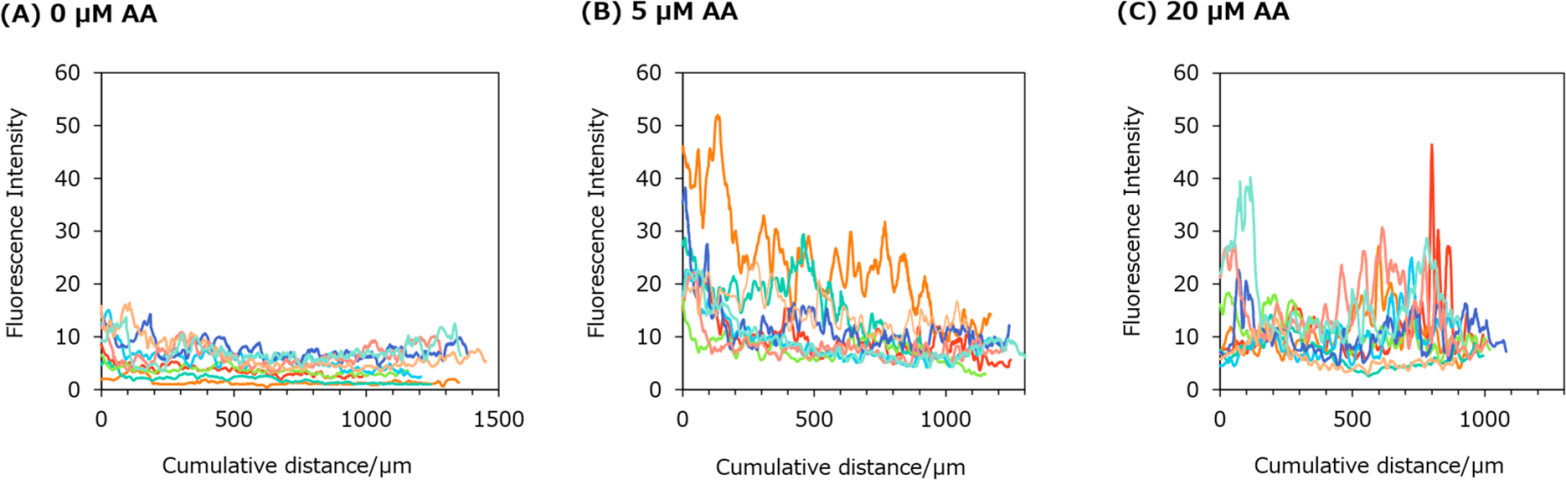
Axial distribution of the fluorescence intensity of dye in the ureter of zebrafish larvae. The horizontal axis represents the cumulative distance along the urinary duct from the glomerulus side to the excretory pore side. The sample size for each group was *n* = 9 for (A) 0, (B) 5, and (C) 20 μM.

The ureteral axial distribution of dye fluorescence intensity in zebrafish larvae exposed to low (5 μM) and high (20 μM) concentrations of AA is shown in Figure 4.

**Figure 5:**
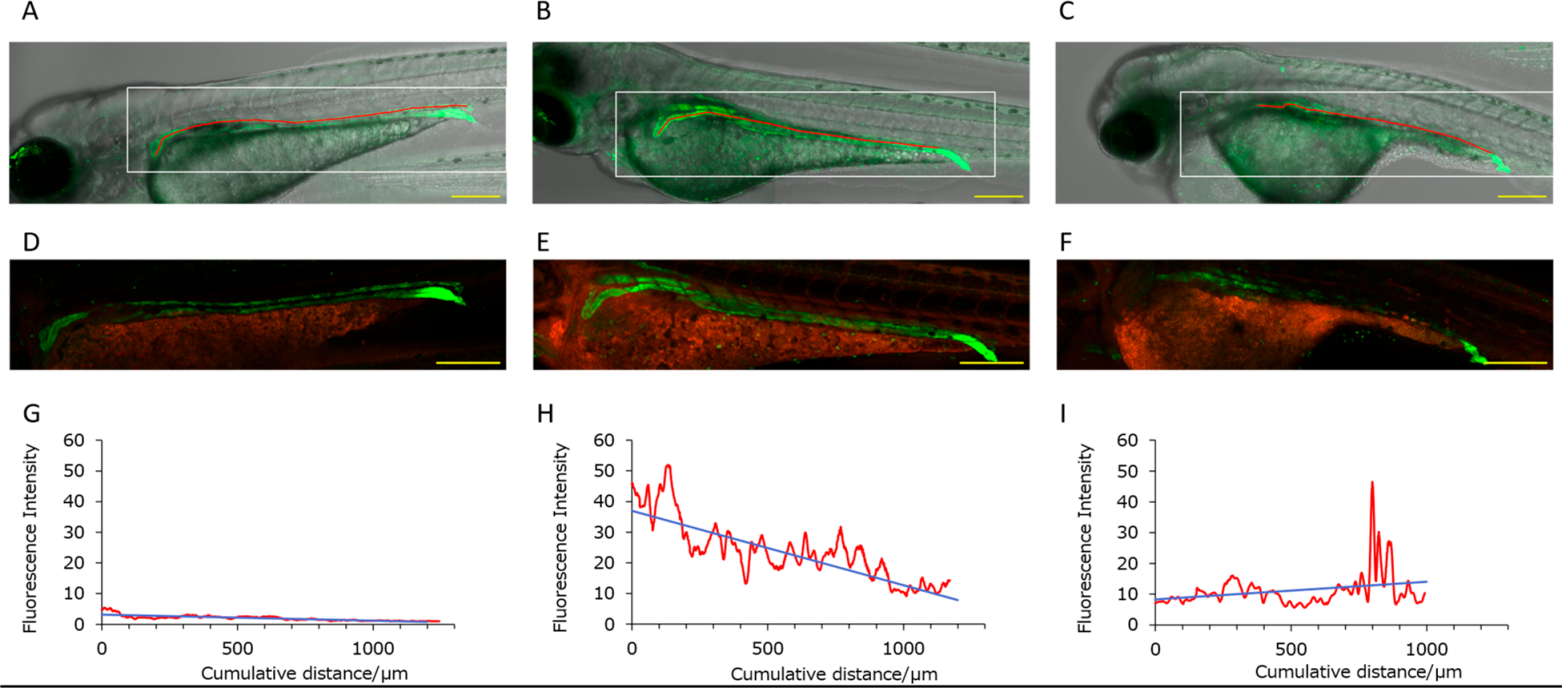
(A–C) Merged bright-field and GFP fluorescence images. The ureter designated based on GFP fluorescence intensity is indicated by the multiple red linear ROIs. (D–F) Merged images of GFP fluorescence image (green) and ZMB741 fluorescence image (red) with the boxed areas in (A–C) enlarged, respectively. The yellow scale bar is 200 μm. (G–I) The transition (red) and approximate straight line (blue) of ZMB741 fluorescence intensity in the ureter. (A, D, G) Examples of individuals without exposure to aristolochic acid. (B, E, H) Examples of individuals exposed to 5 μM aristolochic acid. (C, F, I) Examples of individuals exposed to 20 μM aristolochic acid.

When ZMB741 was continuously exposed to 5 days post fertilization (dpf) larvae of zebrafish at concentrations of 1 μM, it migrated into the bloodstream within 5 min and then accumulated in the gastrointestinal tract by biliary excretion. No significant increase in mortality was observed, even when the fish were left in this state for 3 days. Given that this concentration is not considered to be acutely toxic and that a concentration of 1 μM is sufficient for fluorescent staining of zebrafish blood, ZMB741 was deemed to possess suitable properties as a staining agent for bioimaging applications. The fluorescence intensity within the ureter in the 0 μM AA-exposed group (control group) remained flat. In the 5 μM AA-exposed group, the fluorescence intensity within the ureter was higher near the glomerulus, with a decreasing trend toward the excretory pore. In the 20 μM AA-exposed group, the fluorescence intensity within the ureter remained relatively high from the glomerulus toward the excretory pore. As the body length decreased with increasing AA exposure concentration, the cumulative distance of the pronephric tubule also decreased. In some individuals, there were sudden peaks in fluorescence intensity in parts of the ureter. The sudden peak in fluorescence intensity was indicative of the influx of blood into the ureter (i.e., the hematuric state; see Figure 5).

### Axial Distribution of Dye Fluorescence Intensity in the Ureter of Zebrafish Larvae Using Linear Approximation

**Figure 6:**
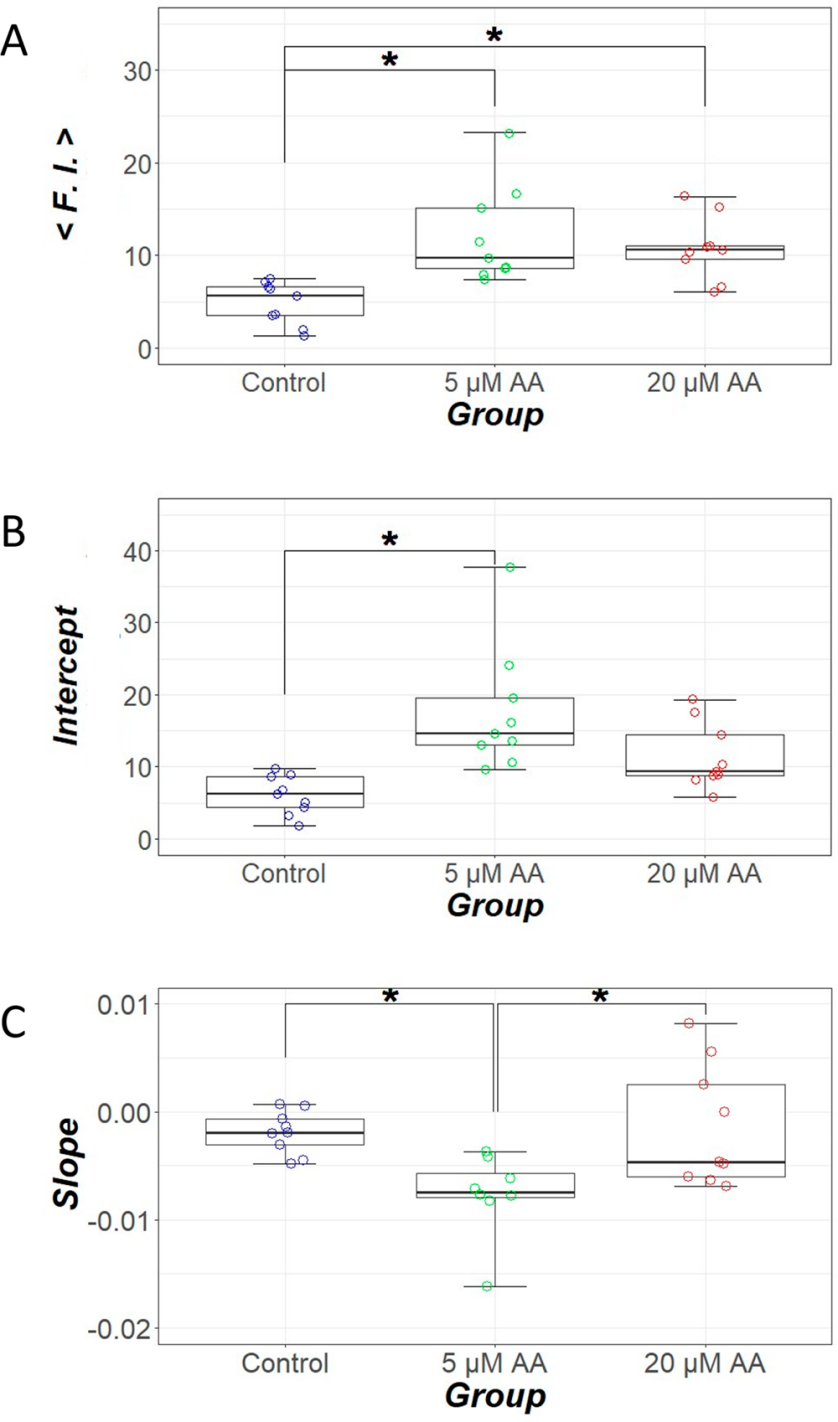
Linear approximation analysis of the axial distribution of dye fluorescence intensity in the ureter of zebrafish larvae. (A) Mean fluorescence intensity. (B) Intercept and (C) slope when the fluorescence intensity is linearly approximated. *P < 0.05.

Figure 6 shows the linear approximation analysis of the axial distribution of dye fluorescence intensity in the ureter of zebrafish larvae. The mean fluorescence intensity ⟨F.I.⟩ significantly increased in both low and high AA exposure groups compared with the control group (*P* = 0.0009 at 5 μM AA and *P* = 0.0025 at 20 μM AA). The intercept of the low AA exposure group significantly increased compared with the control group (*P* = 0.0002 at 5 μM AA and *P* = 0.0525 at 20 μM AA). The slope of the low AA exposure group significantly decreased compared with the control group (*P* = 0.0022 at 5 μM AA and *P* = 0.8589 at 20 μM AA). The slope of the high AA exposure group significantly increased compared with the low AA exposure group (*P* = 0.0135).

**Figure 7:**
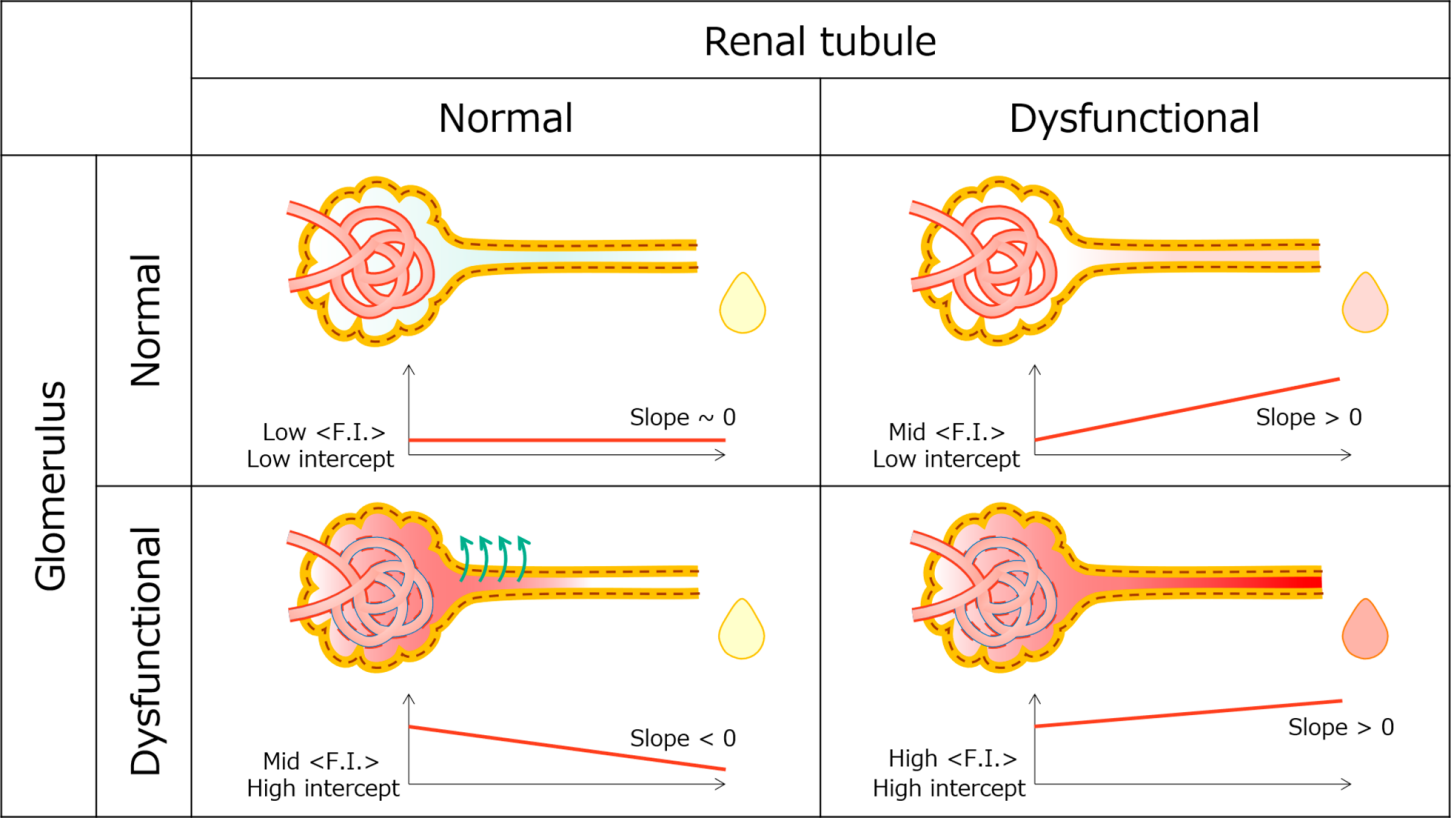
Schematic of differences in fluorescence intensity profiles of ureters based on glomerular and tubular function.

The variation in dye fluorescence intensity distribution due to the AA exposure concentration is linked to the differential impact of AA on the glomerulus and proximal tubules. Figure 7 illustrates different profiles of intratubular dye fluorescence intensity as a function of the glomerular and proximal tubule function. If both glomerular and tubular functions are normal (top left), the fluorescence intensity in the ureter remains low and constant. If the glomerular barrier is impaired and the tubular function is normal (bottom left), the fluorescence intensity curve rises sharply, decreases in slope, and finally decreases toward the exit. In cases where the glomerular barrier is intact and tubular dysfunction (top right) exists, the fluorescence intensity progression starts low, exhibits a positive slope, and finally increases toward the exit. If both the glomerular barrier and tubular function are impaired (bottom right), the fluorescence intensity progression starts high, exhibits a positive slope, and finally increases toward the exit.

The group exposed to a low AA concentration displayed a fluorescence intensity progression similar to that displayed in the lower left of Figure 7, whereas the group exposed to a high AA concentration demonstrated a fluorescence intensity progression resembling that displayed in the lower right. The glomeruli are potentially more sensitive to renal injury from AA compared with proximal tubules.

### Feasibility Study for Drug Discovery Screening

**Figure 8:**
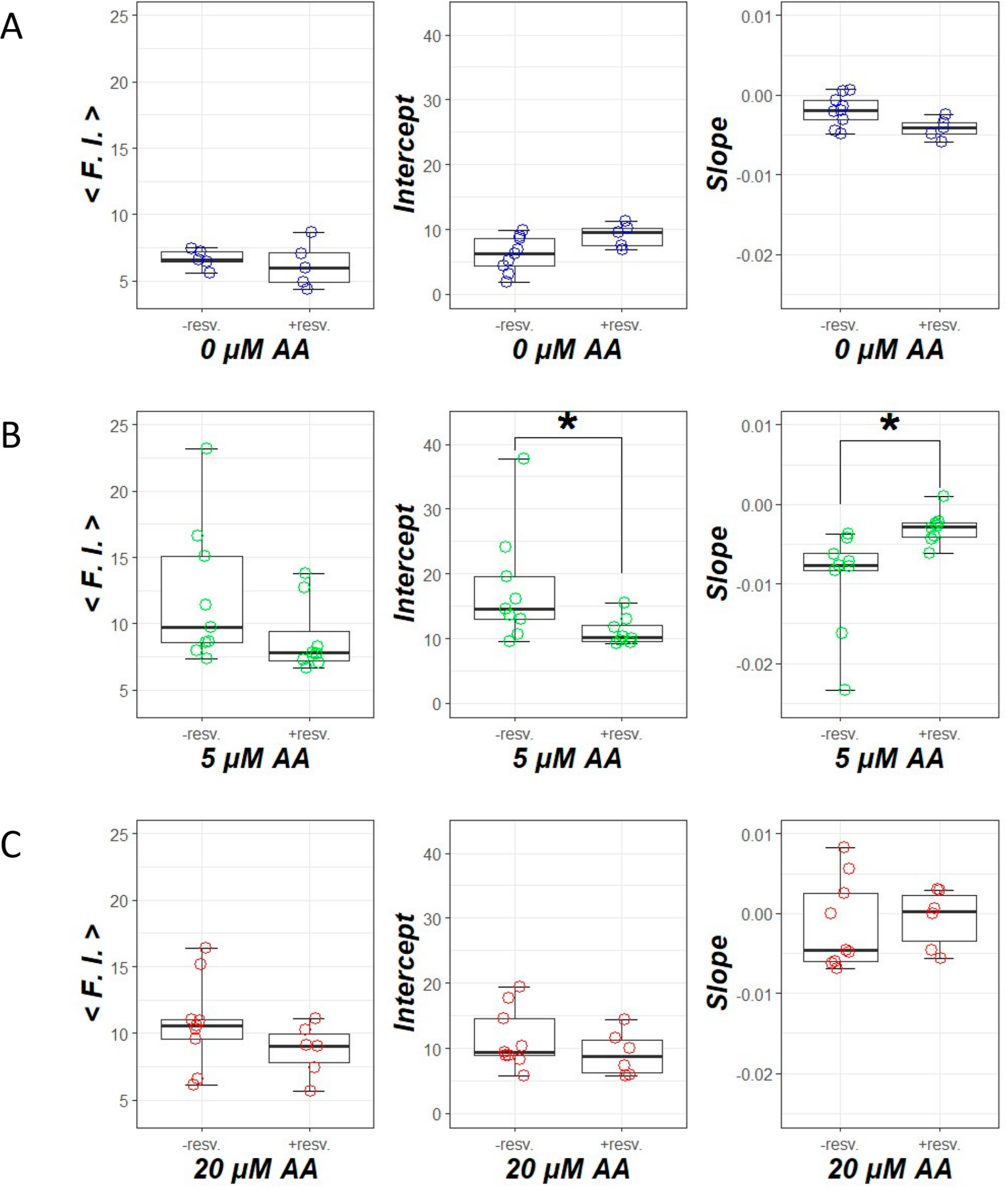
Evaluation of chemical screening methods based on plasma protein leakage into the ureter. (A) The control group without exposure to aristolochic acid. (B) The group exposed to 5 μM aristolochic acid. (C) The group exposed to 20 μM aristolochic acid. In each group, changes in the distribution of plasma proteins in the ureter are evaluated with and without resveratrol administration. *P < 0.05.

Figure 8 provides examples of a chemical screening technique that assesses the amount of plasma protein leakage into the ureter.

Preloading with 90 μM resveratrol before AA exposure can mitigate renal dysfunction, with a more prominent effect observed under low AA concentration conditions.

ZMB741 is a solvatochromic dye that shows almost no fluorescence in aqueous solution; however, its fluorescence quantum yield increases when its conformation is a fixed biomolecule interaction. ZMB741 was initially recognized as a solvatochromic dye that binds to albumin and increases the fluorescence intensity upon binding. It can rapidly enter zebrafish blood via static immersion, facilitating blood fluorescence staining. However, zebrafish lack albumin, and their plasma protein binding target remains unknown. The authors found that commercially available proteins homologous to the major plasma proteins of zebrafish exhibited binding affinity and fluorescence intensity enhancement comparable to those of albumin. Furthermore, ZMB741 can be used to fluorescently stain human plasma proteins developed in native polyacrylamide gel electrophoresis, regardless of their molecular weight (Figure S1). In other words, ZMB741 exhibits low binding target specificity for plasma proteins, has an affinity for a diverse range of plasma proteins, and displays fluorescence enhancement associated with affinity binding. This allows for the visualization of plasma protein leakage in regions where such proteins are typically absent in live fluorescent images. This highlights the potential of applying this concept to the kidney in order to visualize plasma protein leakage into the ureter, where plasma proteins are usually scarce.

This study demonstrated that by using zebrafish pronephroi, one can isolate and evaluate two major functions that support the robustness of hemofiltration in living individuals: molecular sieving in the glomerulus and reabsorption of low-molecular-weight proteins in the proximal tubule. Zebrafish pronephroi allow for the easy spatial localization of these sites. AA induced renal damage of varying severity in zebrafish larvae pronephroi via fertilized egg exposure, displaying an exposure concentration-dependent distribution profile of urinary protein molecular weight. Notably, in this model, individuals exposed to low concentrations (5 μM) of AA exhibited minimal or no increase in the level of protein leakage from the body. Even in individuals exposed to a low concentration (5 μM) of AA, fluorescence enhancement related to plasma protein leakage was observed in the ureter, with the fluorescence intensity decreasing toward the excretory pore. These individuals demonstrated impaired glomerular molecular sieve function, causing plasma protein leakage into the ureter. However, their proximal tubular reabsorption function remained intact and compensated for renal function, enabling the identification of conditions that do not result in the urinary excretion of proteins. Traditional methods using exogenous or endogenous tracers have difficulty detecting such urinary protein signs.

As the assessment of urinary proteins in this study relied on fluorescent images of individuals, it was feasible to examine their correlations with other pathological conditions. For example, as AA affects the heart function of zebrafish,^28^ it enables the investigation of the relationship between renal function assessment and cardiac function through urine protein evaluation and edema severity. Furthermore, it facilitates the examination of the correlation between podocyte-related pathogenesis and proteinuria by visualizing and evaluating glomerular conditions.

The methods utilized to assess urine proteins in this investigation can also be applied in the pursuit of chemicals that potentially hinder the molecular sieve function within glomeruli and the reabsorption capacity of low-molecular-weight proteins in proximal tubules and their corresponding therapeutic compounds. The solvatochromic dye ZMB741 has broad binding selectivity, which is advantageous for the comprehensive visualization of urinary proteins amidst a mixed assembly of plasma proteins. Nevertheless, its lack of specificity precludes the identification of specific urinary protein constituents, indicating the need to investigate the origin of urinary proteins. This approach, which exhibits low specificity and broad binding selectivity, is adaptable to the initial stage of searching for therapeutic agents while minimizing the cost and resource expenditure. Resveratrol may protect the molecular sieve function of glomeruli, particularly when it is impaired by low concentrations of AA.

## Conclusions

ZMB741, a solvatochromic dye, can be used to evaluate proteinuria pathogenesis in zebrafish larvae based on the increased fluorescence intensity of the dye in the pronephros. Fluorescence images acquired while immersed in the dye allowed for a comparison of the distribution of plasma protein leakage into the ureter in multiple individuals, as the dynamics of the dye in the body remain in equilibrium for an extended duration. ZMB741 may be a useful tool for high-throughput chemical screening in a multiwell plate format, with the potential to distinguish glomerular filtration dysfunction from reabsorption dysfunction in the kidney, aiding the study of the pathophysiology of proteinuria in zebrafish.

## Supporting Information Available

Figure S1: fluorescently staining human plasma proteins with ZMB741 (PDF)

## Abbreviations

AA: aristolochic acid I
GFP: green fluorescent protein
ROI: region of interest
