# Supplementary material for: In Vivo Assessment of Individual and Total Proteinuria in Zebrafish Larvae Using the Solvatochromic Compound ZMB741
Source: Chem Biomed Imaging. 2024 May 31;2(11):755–64. doi: 10.1021/cbmi.4c00029 (PMC11600188; doi:10.1021/cbmi.4c00029)
Supplement: Supplementary file 1 — im4c00029_si_001.pdf [file im4c00029_si_001.pdf]

Supporting Information

In vivo assessment of individual and total proteinuria in zebrafish larvae using the solvatochromic compound ZMB741

Tsuyoshi Nomoto[1,2], Aoi Mori[1,2], Kayoko Yamada[1,2], Fumihiro Terami[1,2], Akiyoshi Shimizu[1,2], Toshio Tanaka[1,2,]*

[1]Department of Systems Pharmacology, Mie University Graduate School of Medicine, Tsu, Mie, Japan

[2]Mie University Medical Zebrafish Research Center, Tsu, Japan

***Corresponding Author**

Toshio Tanaka, Department of Systems Pharmacology, Mie University Graduate School of Medicine, Tsu, Mie 514-8507, Japan; E-mail:tanaka@doc.medic.mie-u.ac.jp

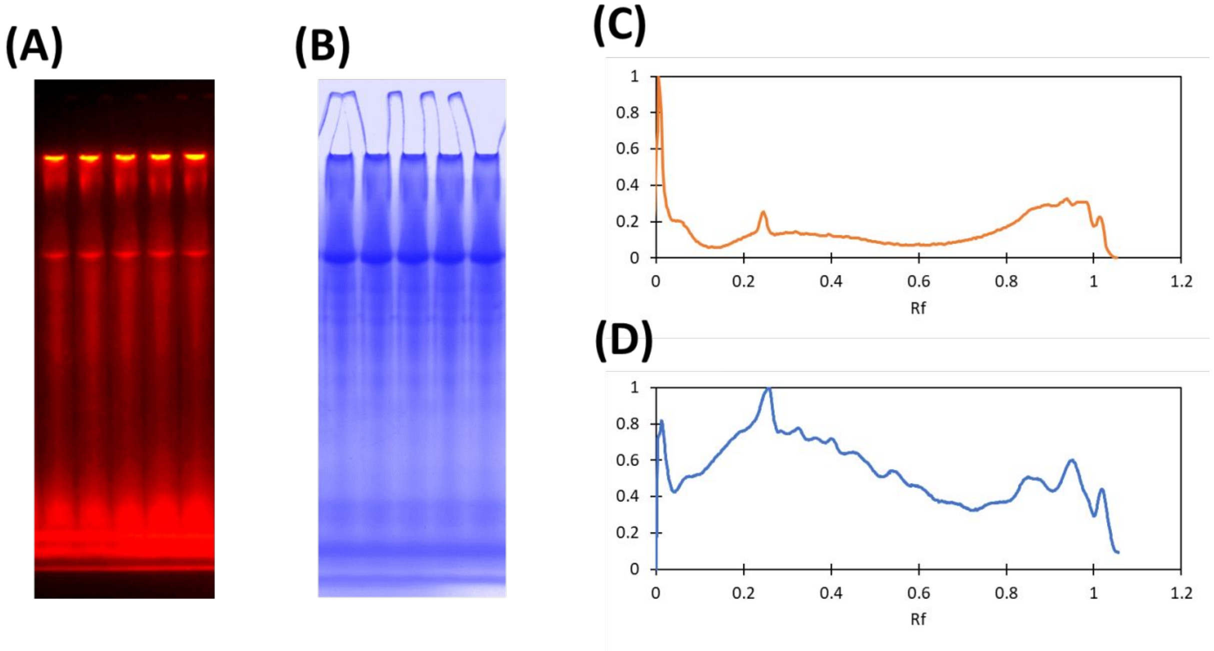

Figure S1. (A) ZMB741-stained fluorescence image of human serum developed using native polyacrylamide gel electrophoresis. (B) Protein-stained image of the gel shown in (A). (C) Fluorescence intensity plotted against the migration rate in (A). (D) Staining intensity compared with the migration rate in (B). Serum was mixed with NativePAGE 4× Sample Buffer (Invitrogen), and a 4 μL per lane equivalent was loaded. NuPAGE of 4%–12% Bis-Tris 1.0 mm Mini Protein Gel (Invitrogen) was used to perform electrophoresis at a constant voltage of 100 V for 24 h at 4 ˚C. Staining of gels with ZMB741 was performed in a 1 μM aqueous solution for 1 h at 25˚C. Fluorescent gel images post-staining were captured using IVIS Lumina LT (PerkinElmer, Inc.).

The identical gels were subsequently stained using Simply Blue Safe Stain (Invitrogen). Protein-stained images were acquired using Odyssey DLx (LI-COR, Inc.). The ImageJ software was used to analyze image intensity relative to migration rate.
